# Statistics

**Published:** 1849-11

**Authors:** 


*Statistics.—Number of Doctors.*—There are in the city of Cincinnati, according to the directory, 179 doctors, good, bad and indifferent. They are classified as follows: Regular physicians, 127; Botanics, (Thompsonian) 8; Eclectics, (branch of the former,) 17; Homœopathists, 10; Indian, 1; unknown, 16. The above enumeration falls considerably short of the actual number.—*Western Lancet.*

